# Exploring Factors Affecting Millennial Tourists’ eWOM Behavior: A Lens of BRT Theory

**DOI:** 10.3390/bs14111056

**Published:** 2024-11-06

**Authors:** Zibin Song, Yingying Ren, Jie Li

**Affiliations:** College of International Tourism and Public Administration, Hainan University, Haikou 570228, China

**Keywords:** reasons for, reasons against, eWOM, consumer behavior, Millennial tourists, behavioral reasoning theory (BRT)

## Abstract

This study employs behavioral reasoning theory (BRT) to investigate factors (i.e., personal values, reasons, and attitudes) affecting existing and future behaviors of Millennial tourists’ electronic word-of-mouth (eWOM). It uses a mixed-methods approach that includes qualitative interviews with 25 tourists to elicit specific reasons for and against eWOM and a survey of 572 Millennial-Chinese tourists to quantitatively validate our BRT structural model. The statistical results from SmartPLS 3.0 show that all hypotheses on direct effects have gained empirical support except for the relationships between the existing behavior and its respective reasons for and against eWOM. These two insignificant direct effects are, however, shown to be fully mediated by global attitudes, respectively. Moreover, gender moderates the relationships between reasons against eWOM and the existing behavior and reasons for eWOM and future behavior, respectively. Most findings regarding the foregoing direct, mediation, and moderation effects are exploratory. In addition, this study contributes significantly to the literature by successfully developing and validating the scale of reasons for and against Millennial tourists’ eWOM within the BRT framework. Destination managers can use this scale of reasons as both a diagnostic tool and a blueprint for eWOM management.

## 1. Introduction

Driven by the advancement of information technology, today’s global tourism industry is expanding rapidly, and competition among destinations is becoming fiercer [1]. One way for a given destination to stay competitive is to better understand its tourist behavior and needs based on tourist data, followed by the development of valid marketing strategies [2]. Destination marketers and managers, for example, frequently use travel-related electronic word-of-mouth (eWOM), which is defined as knowledge exchange that tourists conduct online, to align their efforts with traveler demands [3,4]. While more and more existing tourists share their eWOM information (e.g., posts, videos, photos, and comments on social media), potential tourists then search and review such eWOM information that can either inspire them to book immediately or defer their intentions to visit a place [3]. Ishii and Kikumori [5] estimate that WOM-acquired consumers are twice as valuable as other consumers and that the influence of eWOM on sales is greater than advertising and personal selling combined. Furthermore, both positive and negative eWOM influence consumer purchase intentions and behaviors, as well as product/service assessments [6]. Understanding consumers’ eWOM behavior, as well as its antecedents and consequences, is, therefore, of vital importance for tourism and hospitality businesses [4,7].

A number of hospitality and tourism studies have explored and confirmed eWOM behavior as well as its influencing factors through the lens of at least eleven theories, such as the theory of planned behavior (TPB) and the theory of reasoned action (TRA) [8,9]. A review of the literature indicates, however, that there has been a lack of empirical studies that employ behavioral reasoning theory (BRT) [10] to identify factors affecting consumers’ behavior of eWOM. It also indicates that there has been a lack of measurement scales of reasons for and against eWOM in the tourism and hospitality literature. This limitation, in turn, hampers research and practice in understanding the eWOM behavior of tourists in general and Millennial tourists in particular. The Millennial generation, aged from 18 to 35 [11], is a significant economic force that shapes emerging consumer patterns for one notable reason. Namely, this generation reacts to goods and services via smart technology and the internet at any place and time [12].

Millennial tourists, as per Yousaf et al. [13], account for roughly 23% of all global and domestic travel and have contributed approximately USD 400 billion in 2020, making them an appealing traveler segment. Kim and Park [14] comment that there is less research, however, on how Millennials are shaping and influencing tourism than any other age-related market categories. Thus far, only a few studies (e.g., [15,16]) have examined Millennials’ eWOM behavior as well as its antecedents within the theoretical framework of TRA or TPB, wherein context-specific reasons for and against the behavior, for instance, are neglected [10]. Comparatively, BRT combines traditional constructs (e.g., beliefs, global attitude, intentions, and behavior) from behavioral intention theories (e.g., TRA) with reasons concepts from social sciences [17]. Moreover, BRT argues that context-specific reasons explain incremental variance in behavior after controlling for the influence of individuals’ beliefs and global attitudes [10,17]. The lack of a measurement scale of tourists’ reasons for eWOM further prevents investigators from establishing nomological networks to other BRT theoretical constructs, including personal values, global attitudes, and existing and future behaviors in the tourism context. A review of the literature indicates, for instance, that there has been a lack of empirical evidence on the mediating roles of global attitudes in the relationships between reasons and existing/future behavior of tourists. Potential moderators, such as gender in BRT models, have also not been documented in the literature. These gaps/limitations, in turn, hamper research and practice in understanding and managing tourists’ eWOM behavior.

Based on the foregoing, the overall objective of this study was to employ BRT as our theoretical lens to explore and/or confirm multiple factors that explain the variance in existing and future behaviors (i.e., behavior and behavioral intention) of Millennial tourists’ eWOM. As noted earlier, there has been, however, a lack of a measurement scale of reasons for and against eWOM of Millennial tourists. It is a must to develop and validate this scale of reasons prior to realizing the overall research objective. Specifically, the study poses the following specific objectives:

(a) To develop and validate a measurement scale of reasons for and against eWOM of Millennial tourists;

(b) To explore and/or confirm proximal (i.e., attitudes and reasons) and distal (self-enhancement) antecedents for eWOM behavior of Millennial tourists;

(c) To detect the mediation roles of global attitudes in the relationships between reasons and existing/future behaviors of Millennials’ eWOM;

(d) To explore the potential moderation role of gender in the relationships between reasons and existing/future behaviors of Millennials’ eWOM.

By realizing the foregoing research objectives, this study is very likely to contribute to the body of literature in two ways. One is that most findings regarding the foregoing direct, mediation, and moderation effects are exploratory and thus valuable. The other is that the development and validation of a measurement scale of reasons for Millennials’ eWOM behavior is original and valuable, particularly in consideration of the fact that there has been a lack of such a scale in the literature. In practice, destination marketers and managers could use our measure of reasons as both a diagnostic tool and a blueprint for eWOM management.

## 2. Theoretical Background and Hypotheses

### 2.1. Behavioral Reasoning Theory (BRT)

BRT, proposed by Westaby, provides a logical explanation by distinguishing between the fundamental reasons underlying an individual’s behavior and the psychological processes that influence it [18]. BRT examines the influences of reasons, as well as their interplay with other antecedents (e.g., values/beliefs, attitudes), on existing and future behaviors [17]. In fact, BRT is built on, but not limited to, other traditional theories, including TRA and TPB, and thus offers unique advantages. Specifically, BRT asserts that consumers’ cognitive processing behavior is motivated by reasoning, with two distinct aspects: reason for and reason against a given behavior [17,19]. It explains variance in people’s intentions that go beyond what is captured by traditional models, such as TRA [19]. Moreover, BRT emphasizes the context-specific nature of values/beliefs and reasons for and against the behavior, providing a nuanced and notable perspective on consumer behavior [20]. Therefore, BRT is gaining increasing attention in the domain of individual behavior.

Thus far, BRT has been used in the context of leadership behavior [21], renewable energy innovation [19], organic food consumption [22], new technology adoption [20,23], pro-environmental behavior [24], and fake news-sharing [25], among others. However, only a few tourism and hospitality studies relevant to eWOM have used BRT as their theoretical framework. Berné Manero et al. [26], for instance, delve into the process by which hotel managers make decisions regarding the adoption and implementation of eWOM as a management strategy. More often than not, tourists’ behaviors are documented in empirical works (e.g., [8,9]) and are captured through the lens of traditional behavioral theories (e.g., TPB). Moreover, existing research (e.g., [8,15]) on tourists’ WOM often focuses on their reasons for eWOM behavior while neglecting their reasons against the same behavior. Hence, the current study employs BRT to gain a deeper understanding of the process through which tourists make decisions to engage in eWOM behavior. Additionally, it addresses the recommendation by Sahu et al. for more comprehensive research on BRT across various contexts [17].

In the BRT context, values are cross-situational goals that guide an individual’s life [27]. Reasons are viewed as specific subjective factors used to explain intended behavior [10]. Attitude is defined as a person’s overall positive or negative evaluation of a particular behavior [10,28]. In the literature, behavior is also referred to as existing behavior, while behavioral intention is also known as future behavior, which is defined as the individual’s propensity to engage in a specific behavior or task [23].

### 2.2. Reasons for, Reasons Against, Attitude, and Existing and Future Behavior

When considering reasons for or against a particular behavior related to an object, people form their attitude based on readily available thoughts, potentially resulting in a shift in their attitude toward that object [24]. Westaby [10] argues that the reasons for/against a behavior strongly impact one’s attitude as they are employed to justify, defend, and maintain one’s decisions and judgments, ultimately mirroring and embodying one’s attitude. In other words, when individuals present reasons for their behavior toward an object, which stem from their overall attitude toward it, this reinforces their attitude as a result [29]. Conversely, when an individual evokes a reason against a behavior, the strength or intensity of the attitude is diminished.

Scholars have validated the relationship between reasons and attitudes in multiple fields (e.g., [19,23,24,30]). In particular, Pillai et al. [23] examine the connection between attitudes and the reasons for/against them in the context of the adoption of AI-based chatbots. Their findings indicate that positive attitudes may arise from reasons like personalization and interactivity, whereas negative attitudes may be prompted by concerns related to perceived risk and technological anxiety. These reasons, in turn, reinforce and mold individuals’ attitudes toward adoption, ultimately impacting their decisions and behaviors within this domain. Transferring this idea to the situation of Millennial tourists’ eWOM, we therefore develop the following hypotheses:

**H1a/b:**  *Reasons for (H1a) and against (H1b) eWOM influence Millennial tourists’ attitudes toward eWOM, respectively*.

From the perspective of BRT, reasons play a significant role in motivating intentions as people feel comfortable when they have sufficient reasons to justify their intended actions [10]. Individuals employ reasons to comprehend the world and rationalize their behavioral decisions, which helps them avoid discomfort or inconsistency [31]. In particular, Westaby [10] argues “reason for” to drive behavior, while its counterpart of “reason against” to inhibit the same behavior. Reasons encompass the justifications and defensive mechanisms that, consequently, shape behavioral intentions, surpassing the scope of explanations provided solely by beliefs and attitudes [18]. This is because individuals are inclined to select options, even if they are unfavorable and can be readily rationalized [24]. In addition, people often simplify decision-making by using cognitive shortcuts [10]. These theoretical notions have received supportive empirical evidence across a variety of contexts, such as mobile banks and new application adoption [20,32]. Transferring this idea to the context of Millennial tourists’ eWOM, we thus develop the following hypotheses:

**H2a/b:**  *Reasons for (H2a) and against (H2b) eWOM influence Millennial tourists’ existing behavior of eWOM, respectively*.

**H2c/d:**  *Reasons for (H2c) and against (H2d) eWOM influence Millennial tourists’ future behavior of eWOM, respectively*.

Consumers’ attitudes positively influence their behavior [33]. Attitude reflects an individual’s overall assessment of the usefulness or lack of usefulness of performing a particular action based on their evaluation of the consequences of the behavioral reasoning process [34]. The relationship between attitudes and behavior has been extensively researched and documented in many works [20]. Jabeen et al. [35] provide supportive evidence that individuals with a negative attitude toward food waste are more likely to reduce waste. Moreover, the relationship between tourists’ attitudes and behavioral intentions has been substantiated in the context of heritage tourism attractions [36] and South Korea as a tourism destination [37]. Picazo-Vela et al. [38] also report that attitude has a significant impact on the intention to write online reviews. However, Dixit et al. [34] fail to reveal the relationship between attitude and behavioral intention in the context of writing online reviews. Moreover, Hung et al. [39] find that attitude toward knowledge sharing significantly influences intention. Based on the foregoing, the research could be extended to the context of Millennial tourists’ eWOM. We therefore develop the following hypotheses:

**H3a/b:**  *Millennial tourists’ attitudes toward eWOM influence their existing (H3a) and future (H3b) behaviors of eWOM*.

### 2.3. Value, Reasons for, Reasons Against, and Attitude

Schwartz et al. [27] argue that human values may manifest themselves, in a given situation, as self-enhancement and openness to change, among others. Values are persistent beliefs related to behaviors [40]. In the present study, value is operationalized as self-enhancement for a notable reason. Namely, self-enhancement involves pursuing personal interests and improving self-concept (e.g., [27]), and individuals with these values usually prefer sharing activities (e.g., [41]). In the context of tourist eWOM behavior, Millennial tourists’ self-enhancement is likely to influence their reasons for or against eWOM behavior. This is because self-enhancement functions as a strong and internal engine that forces one to develop and uphold a favorable self-view in a variety of domains, including perception, memory, and experiences [42]. This notion echoes one important BRT proposition, namely, value serves as a critical precursor to the reasons individuals use to justify and support their anticipated behavior [10]. In the same vein, expectancy-value theory suggests that values impact motivations, informing reasons for and against a behavior during decision-making [43]. In fact, scholars have validated the relationship between value and reasons in multiple fields (e.g., [10,24,32,44]). Based on the foregoing, the research could be extended to the context of Millennial tourists’ eWOM. We therefore develop the following hypotheses:

**H4a/b:**  *Value (self-enhancement) influences Millennial tourists’ reasons for (H4a) and against (H4b) eWOM, respectively*.

According to Westaby [10], people’s values are theoretically expected to have a direct influence on their attitudes. Instead of relying on reasons that are not highly active in decision-making, consumers may turn to heuristic motives [10]. Unlike attitudes or beliefs, values form an organized system and are generally considered to shape attitudes and behaviors [31]. Empirically, previous research indicates that values affect attitudes in many contexts (e.g., [44,45,46]). For example, Qian et al. [46] reveal that value significantly impacts ethical attitudes within the context of autonomous vehicle-hailing services. It has also been shown that individual values reflecting consumers’ perception of luxury have a positive impact on attitudes toward jewelry purchasing behavior [31]. Based on the foregoing, the research could be extended to the context of Millennial tourists’ eWOM. We therefore develop the following hypothesis:

**H5:**  *Value (self-enhancement) influences Millennial tourists’ attitudes toward eWOM*.

### 2.4. The Mediating Role of Attitude

According to the BRT [10,17], reasons directly predict future behavior and also mediate the path between reasons and future behavior through attitude. Consumer behavior research has shown that several factors influence attitudes, which then translate into behavioral intentions [35]. Attitudes are key mediators of relationships in different contexts. For example, Tandon et al. [47] explore the mediating role of attitude toward purchasing organic food in the relationship between reasons (for and against) and purchase intention. Jabeen et al. [35] indicate that attitude plays a significant mediating role in the relationship between emotion and future behavior in reducing food waste. Sharma et al. [48] suggest the mediating role of attitudes in the relationships between reasons and future behavior, for example. However, some studies (e.g., [44]) indicate that attitudes do not act as mediators. Based on this, we developed the following hypotheses:

**H6a/b:**  *Millennial tourists’ attitudes toward eWOM mediate the relationships between reasons for (H6a) and against (H6b) eWOM and their existing eWOM behavior, respectively*.

**H6c/d:**  *Millennial tourists’ attitudes toward eWOM mediate the associations between reasons for (H6c) and against (H6d) eWOM and their future eWOM behaviors, respectively*.

### 2.5. Gender as a Moderator

In fact, many scholars (e.g., [49,50]) underscore the importance of gender as well as its moderating effect on consumers’ decision-making processes and outcomes. Gundala et al. [51], for example, argue that males and females have different values, social expectations, and behaviors because, in their early childhood, they have experienced different socialization processes. According to gender socialization theory [52], the differing socialization processes undergone by males and females result in behavioral differences. Men tend to be more self-focused, independent, and decisive, whereas women are more concerned about themselves and others, less independent, and more susceptible to influence [53]. As such, eWOM behaviors could be seen as a form of social interaction [54], where tourist behaviors between male and female tourist groups are reasonably expected to be influenced differentially. A review of the literature indicates that there has been a lack of gender-moderating roles in shaping tourists’ eWOM behavior/behavioral intention; we, therefore, develop the following hypotheses:

**H7a/b:**  *Gender moderates the relationships between reasons for (H7a) and against (H7b) eWOM and existing behavior in that these relationships will be stronger for a female group than for a male group*.

**H7c/d:**  *Gender moderates the relationships between reasons for (H7c) and against (H7d) eWOM and future behavior such that these relationships will be stronger for a female group than for a male group*.

## 3. Methodology

### 3.1. Measurement Scales for Value, Attitude, and Existing and Future Behaviors

Among the BRT factors, self-enhancement, attitudes, and behavioral intention have corresponding existing measures, which are detailed in Appendix A. In particular, we adapted four items developed by Lee et al. [41] to measure self-enhancement using a 5-point Likert scale ranging from strongly disagree to strongly agree. We used a 4-item scale developed by Hung et al. [39] to measure Millennial tourists’ global attitudes using a 7-point Likert scale ranging from strongly disagree to strongly agree.

With regard to future eWOM behavior, five items were adopted, among which three were taken from Šegota et al.’s [55] work, one was contributed by Kim and Hwang [56], and the rest were contributed by Ma et al. [57]. The respondents were requested to indicate the extent to which they would talk positively or negatively about a destination with others on social media platforms. The scale ranges from 1 “negatively” to 7 “positively”. Finally, two items for existing eWOM behavior are developed in this study (detailed in Appendix A).

### 3.2. Steps in Developing the Measurement Scale of Reasons

Given the context-specific nature of the reasons, there has been a lack of measurement scales for reasons for and against eWOM behavior in the literature. We therefore took, as per DeVellis and Thorpe [58], eight steps to develop the measurement scale of reasons for and against eWOM behavior. The first three steps concern the construct’s definition and item pool (detailed in Section 3.2.1). Steps four and five involve experts’ evaluations of the item pool and subsequent revisions on the pool (Section 3.2.2). Steps six and seven concern the empirical validation and evaluation of the proposed scale of reasons (Section 3.2.3). Finally, step eight involves cross-validation and final decisions on the measurement scale (Section 3.2.4 and Section 3.2.5).

#### 3.2.1. Reasons’ Definition and Item Pool

The reasons construct, as per Westaby [10], is context-specific, and therefore, in this study, it could be defined and operationalized as Millennial tourists’ subjective perceptions and evaluations of factors/attributes that strengthen or weaken their decision on eWOM engagement. The qualitative approach was used, and it involves the utilization of methods for collecting qualitative data, such as observation, interviews, and document reviews [59]. In the present study, the pool of measurement items was developed using the following three procedures: semi-structured interviews, the Delphi method, and experts’ review of the content validity of the proposed item pool.

First, semi-structured interviews were conducted to develop an item pool of reasons. The purpose of the interviews was to determine how the participants felt about sharing their experiences and what drove or discouraged them from doing so. All respondents were Millennial visitors who had visited a destination within the past three months. The specific questions are as follows: (1) Have you ever disseminated information about your travel on social media platforms? How many times have you shared? (2) Would you be willing to praise the destinations on social media platforms? And why? (3) Would you be reluctant to share travel experiences or condemn the destinations on social media platforms? And why? As a result, every interview was recorded and lasted for about five to ten minutes. According to Glaser and Strauss [60], data saturation was reached after 25 interviews because no new major themes surfaced in the remaining interviews.

Second, this study adopted the modified Delphi Method [61] to analyze the foregoing qualitative data. Specifically, five experts were asked to listen to the interview records and to identify keywords/codes for the dimensions of both reasons for and reasons against. In case different codes were suggested among the experts, the moderator then gave feedback to corresponding experts, respectively, inviting them to reconsider the codes. Consequently, the pool of reasons has 34 measurement items, among which 17 are tailored for reasons for, and the rest are tailored for reasons against (Appendix B).

#### 3.2.2. Experts’ Evaluations and Subsequent Revisions

Twenty experts (i.e., eleven professors and nine industry managers) were invited to evaluate the content validity of the measurement pool of reasons. They were requested to respond to a 5-point Likert scale ranging from “1” (very unrepresentative) to “5” (very representative). Results (Appendix B) indicate that among the 34 items, 30 items have exhibited their content validity because their mean values are above 3.0, the threshold level suggested by Fetscherin and Stephano [62]. The rest of the 4 items were deleted due to their mean values being below the same threshold level. To compensate for this, 4 supplementary measurement items resulting from a thorough literature review were added to the measurement pool of reasons (Appendix C).

#### 3.2.3. Empirical Validation and Evaluation on the Proposed Scale of Reasons

We then took a quantitative approach to validate and evaluate the foregoing scale of reasons and other BRT scales, including self-enhancement, attitude, existing behavior, and future behavior, among others. A self-reported questionnaire was distributed online in 21 March and 8 May 2024 via Credamo (https://www.credamo.com/), an online paid survey platform in China. Many scholars (e.g., [46]) have used this online data. Moreover, Credamo enables investigators to screen out unengaged participants. As a result, 572 copies of usable questionnaires were collected.

***EFA* *Result of* *Reasons.*** We then divided the overall sample with 572 respondents into two split samples, in accordance with DeVellis. The subsample 1 (N = 287) was used for EFA (exploratory factor analysis) to explore the underlying dimensions of reasons. The EFA, enabled in SPSS 26.0, was based on principal axis factoring with oblique rotation to capture the dimensions [63]. Consequently, a six-factor structure with 27 items was identified, explaining 60.069% of the variance. The six factors are (a)accessibility and cuisines (4 items), (b) happy feelings (3 items), (c) memorable travel experiences (5 items), (d) natural and cultural attractions (2 items), (e) service failures (10 items), and (f) side effect of sharing (3 items). While the first four dimensions capture the reasons for the behavior, the remaining two dimensions snatch reasons against the same behavior (see details in Appendix D).

***CFA Results of Reasons.*** Based on the results of EFA, subsample 2 (N = 285) was used for CFA (confirmatory factor analysis) and enabled in AOMS 24.0. CFA was conducted to verify the dimensions derived from EFA [63]. As a result, the model exhibits, as per Hair et al. [64], well-acceptable levels of fit indices: *χ*^2^*/df* = 1.627, RMR = 0.035, RMSEA = 0.047, CFI = 0.935, IFI = 0.936, and TLI = 0.927. We then took a step further to explore whether a second-order factor solution for the reasons scale fit the data well in the same split sample. Regarding the expectation of BRT, *reasons for* and reasons *against* eWOM each present itself as a second-order factor. This factorial solution for the reasons scale also fits the data well: *χ*^2^*/df* = 1.591, RMR = 0.048, RMSEA = 0.046, CFI = 0.933, IFI = 0.934, and TLI = 0.931. Finally, it should be noted that we consider reasons for and reasons against as formative constructs rather than reflective ones. This is in line with other BRT empirical studies, such as the one contributed by Ashfaq et al. [65].

#### 3.2.4. Development of the Overall Measurement Model of BRT

On the one hand, the overall measurement model enables us to examine the nomological validity of the reasons scale, and on the other, it enables the investigator to examine the psychometric properties of the overall model. In particular, we developed an overall measurement model that includes four first-order factors (i.e., self-enhancement, attitude, behavior, and behavioral intention) and two second-order factors of reasons for and reasons against the behavior. This model fits the data well: *χ*^2^*/df* = 1.770, RMR = 0.034, RMSEA = 0.037, CFI = 0.932, IFI = 0.933, and TLI = 0.927. Comparatively, this model generally fits the data worse than its competing model: *χ*^2^*/df* = 1.817, RMR = 0.031, RMSEA = 0.038, CFI = 0.963, IFI = 0.964, and TLI = 0.956. In the competing model, everything is identical to the foregoing model except for the reasons for and against the construct. In the competing model, 4 factors of reasons for the behavior and 2 factors of reasons against the same behavior are aggregated and averaged into 4 indicators of reasons for the behavior and 2 indicators of reasons against the behavior, respectively. In other words, we used the item parceling method, suggested by Bandalos [66], to measure reasons for and against the behavior in a more parsimonious manner with fewer measurement errors. In addition, item parceling is also used in other BRT studies (e.g., [67,68]). All things considered, we choose the foregoing item parceling model as our final overall measurement model of BRT.

#### 3.2.5. Cross-Validation of the Overall Measurement Model of BRT

To examine the generalizability of the overall measurement model, the foregoing overall measurement model of BRT was cross-validated in split samples 1 and 2. As a result, this overall model manifests itself as being factorial invariance—Δχ^2^ [15] = 15.49, *p* = 0.584—across the foregoing two split samples. In this regard, DeVellis and Thorpe [58] argue that showing factorial invariance across samples is one of the most effective ways to show the generalizability of the foregoing factor structure.

## 4. Results

### 4.1. Demographics

The foregoing overall measurement model has been based on the overall sample of 572 respondents. The sample includes respondents from all provinces and regions in Mainland China, except Ningxia and Tibet autonomous regions. Table 1 shows that over half (55.6%) of the respondents’ ages were between 24 and 29. Likewise, over half (56.6%) of them were fully employed. While 35.8% of respondents were women, 64.2% were men. Most of the respondents (88.1%) had education in college or above.

### 4.2. Psychometric Property of the Overall Structural Model

**Figure 1:**
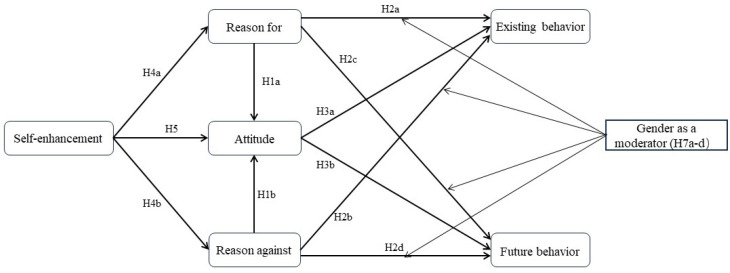
The framework model.

***SEM* *Fit* *Index.*** Figure 1 depicts the overall structural model whereby gender is treated as both a moderator and control variable, a kind of practice that can also be found in Tang’s [69] work. This overall structural model has been estimated in SmartPLS 3.0. This study has employed the Partial Least Squares-Structural Equation Modeling (PLS-SEM) approach, a widely utilized tool for exploring emerging research trends and constructing a model rather than merely confirming existing ones [70]. This methodology effectively addresses the constraints within the constructs by measuring both reflective and formative constructs at the same time [71]. As a result, SRMR values for the saturated and estimated structural models are 0.036 and 0.040, respectively, all smaller than 0.080. This indicates, as per Hair et al. [72], that the overall structural model exhibits acceptable levels of the SEM fit index.

***Constructs’* *Reliability and* *Validity.*** The estimation results enabled in SmartPLS 3.0 show that all six latent constructs have acceptable levels of reliability and validity. Table 2 indicates that composite reliability values range from 0.812 to 0.911, and AVE values vary from 0.516 to 0.836, all higher than, according to Hair et al., the threshold level of 0.70 and 0.50, respectively. This would suggest that all BRT constructs in this study have achieved convergent validity. The discriminant validity of these constructs has also been achieved successfully for one notable reason. Namely, the squared AVE values of these latent constructs vary from 0.718 to 0.914, each of which is higher than any of the correlation values, as shown in the horizontal or vertical cells in Table 3. This would suggest, as per Hair et al. [64], that six latent BRT constructs all have achieved discriminant validity successfully.

***Collinearity* *Statistics (VIF) and* *Common* *Method* *Variance (CMV).*** In this overall model, VIF values of the manifested variables are between 1.00 and 1.82, all smaller than the threshold level of 5.00. This would suggest, as per Hair et al. [72], that collinearity is not an issue in this overall model. Furthermore, the VIF values are smaller than 3.30. This fact reveals that, as per Kock [73], CMV issue does not substantially lead to those estimation values in this overall model.

### 4.3. Hypothesis Testing Results and R Square Values

**Figure 2:**
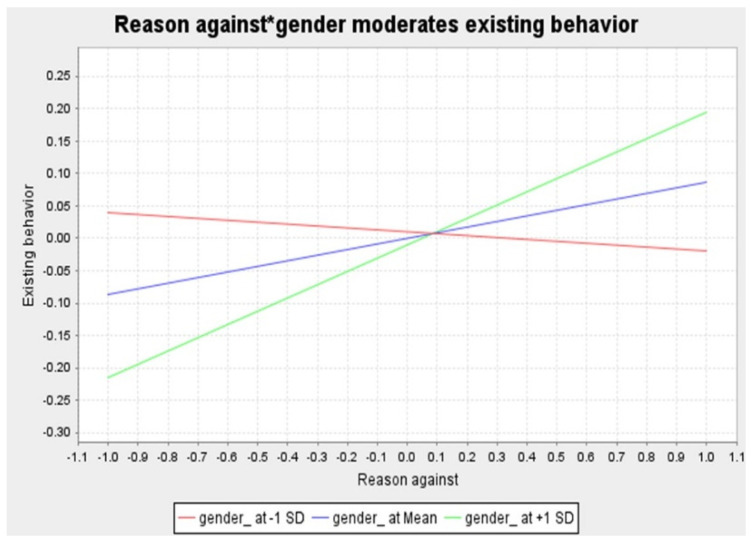
Gender moderates the relationship between reasons against and existing behavior.

**Figure 3:**
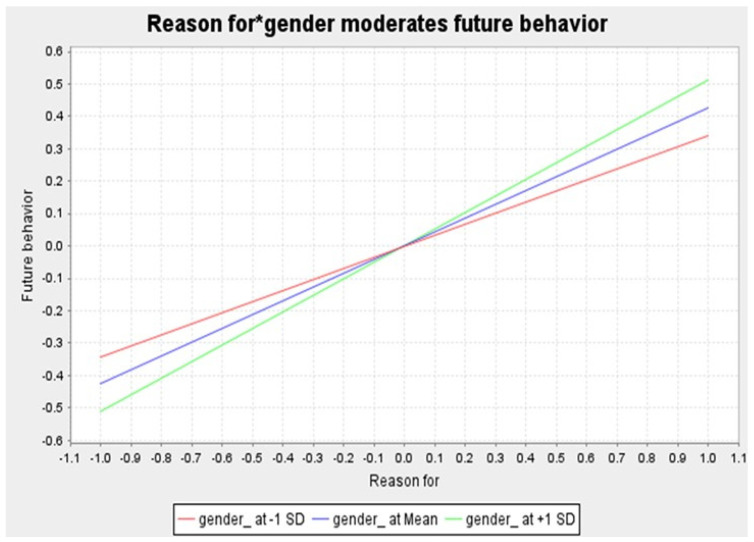
Gender moderates the relationship between reasons for and future behavior.

To evaluate the research hypotheses, we created 5000 bootstraps enabled in SmartPLS 3.0 with 572 parent respondents. Table 4 shows that all hypotheses on direct relationships (H1a to H5b) have received empirical support, except for H2a and H2b. It also reveals that the mediation hypotheses (H6a to H6d) have all gained supportive evidence. While two moderation hypotheses (H7a and H7d) have not been substantiated, the other two moderation hypotheses (H7b and H7c) have otherwise gained empirical support in this overall sample (see Table 4, Figure 2 and Figure 3 for more details).

In the overall structural model, the squared multiple correlation coefficient (SMC) of global attitude is 0.437. SMC values of future and existing behavior are 0.574 and 0.178, respectively. Finally, SMC values of reasons for and against eWOM are 0.173 and 0.050, respectively.

## 5. Discussion and Conclusions

The primary goal of this study was to investigate the multivariate influences of self-enhancement, attitude, reasons for and against eWOM, as well as the interplays between these four BRT antecedents in predicting Millennial travelers’ eWOM behavior. Most of our hypotheses have generally received empirical support in our study. Generally, we contribute to the body of literature in two notable ways. One involves the substantiated direct, mediation, and moderation effects depicted in Figure 1, in that most of them are essentially exploratory in terms of capturing the eWOM behavior of Millennial tourists. The other concerns the development and cross-validation of the measure of Millennial tourists’ reasons for and against eWOM behavior within the BRT framework. In the Sections that follow, we will discuss our contributions/originalities in greater detail.

### 5.1. Originalities and Theoretical Implications

***Contributions of* *Direct* *Effects.*** Generally, nine hypotheses on direct effects (i.e., H1a, H1b, H2, H3a, H3b, H4c, H4d, H5a, H5b) have all gained empirical support in this study. These findings are all consistent with the BRT on the one hand, and on the other, they may be replicative and/or exploratory depending on specific situations. In terms of H2c, the present study provides the first-ever empirical evidence for this linkage in the tourism literature. In BRT literature, this is consistent with some empirical works, such as the one contributed by Qian et al. [46], in terms of future behavior in using autonomous vehicle-hailing services. It contradicts, however, other BRT empirical works, such as the one contributed by Virmani et al. [74], in the context of future behavior of adopting Industry 4.0. Turning to the substantiated H2d, our study provides the *first-ever* empirical evidence to the best of our knowledge of the tourism literature. In BRT literature, this finding contradicts the corresponding findings contributed by Virmani et al. [74]. Conversely, it is consistent with the corresponding finding reported by Mobarak et al. [32] in the context of continuous future behavior of using mobile payment services. While the finding regarding the substantiated H3a is exploratory, the one associated with the substantiated H3b is replicative in the tourism literature because Nieves-Pavón et al. [9] reveal that tourists’ global attitudes positively affect their future behavior of eWOM.

Furthermore, findings regarding H1 are consistent with BRT, and they are exploratory in the tourism literature. Again, this is attributable to the fact that we provide the first-ever measurement scale of tourists’ reasons for eWOM as well as findings obtained by using this scale. H4 is exploratory largely due to the fact that we provide, to our best knowledge, the first-ever measurement scale of Millennial tourists’ reasons for eWOM. Similarly, H5 has gained empirical support in our study, extending the literature substantially because Chandra [75] documents, for instance, that self-enhancement has a positive influence on individuals’ attitudes toward eWOM in the context of technology acceptance.

***Understanding* *Insignificant* *Direct* *Effects.*** In our empirical data, H2a and H2b have failed to be supported. Although these facts are somewhat unexpected and surprising, they are somehow reasonable for one notable reason. That is, the direct effects between reasons and the existing behavior of eWOM have disappeared in the presence of attitude, the mediator proposed in BRT. In fact, this supposition has gained support in that in the absence of global attitude, reasons against eWOM otherwise predict, for instance, the future behavior of eWOM (β = −0.131, *p* = 0.016). Based on the foregoing, it would be suggested that the effects of Millennial tourists’ reasons for and against eWOM on their existing behavior of eWOM are indirect (via global attitude) rather than direct. These findings drop theoretical implications such that global attitude may not always present itself as a partial mediator; rather, it is very likely to be a full mediator in the relationship between reasons and future behavior in the population of, for example, Millennial tourists.

***Originalities of* *Mediation* *Effects.*** In our study, H6 expects that reasons for and against eWOM have indirect effects on both existing eWOM behavior (H6a, H6b) and future eWOM behavior (H6c, H6d), respectively, via global attitude. All these hypotheses have gained empirical support in this study. In the tourism literature, all the findings are exploratory. In BRT literature, findings regarding H6c and H6d are consistent with the corresponding indirect effects in Tandon et al.’s [47] work in terms of future behavior of organic food purchases. Meanwhile, they contradict, however, the corresponding indirect effects between reasons and future behavior of organic food purchases reported in Thi Nguyen and Dang’s [44] work wherein global attitude is not a substantiated mediator. Comparatively, the findings regarding H6a and H6b are very original because existing behavior, as well as reasons’ indirect effect on this behavior via attitude, has not been documented elsewhere in the literature. Moreover, most BRT empirical works (e.g., [4,23,32]) only examine the direct effects among latent theoretical constructs, neglecting the assessment of indirect effects with only a few exceptions (e.g., [44,47]) that look at both. Based on the foregoing, it could be stated that the mediation effects identified in this study contribute significantly to the literature.

***Originality of* *Moderation* *Effects.*** As shown in Table 4, gender does not moderate the relationship between reasons for eWOM and existing behavior of eWOM. It moderates, however, the linkage between reasons against eWOM and the existing behavior of eWOM. Post-hoc group regression analysis reveals that reasons against significantly impact female Millennial tourists’ existing behavior (β = 0.211, *p* < 0.05), but not that of male Millennial tourists (β = −0.016, *p* > 0.1). Likewise, gender fails to moderate the relationship between reasons against eWOM and the future behavior of eWOM. It significantly moderates, however, the relationship between reasons for eWOM and the future behavior of eWOM. Post-hoc group regression result indicates that female Millennial tourists’ future behavior is more affected by reasons for (β = 0.511, *p* < 0.001) than that of male Millennial tourists (β = 0.345, *p* < 0.001). The foregoing moderation effects are reasonable given the fact that Šerić et al. [76], for instance, document that the effect of destination reputation on destination residents’ WOM is stronger for women than for men. The foregoing moderation effects regarding Millennial tourists are not documented elsewhere in the literature except for this study. As such, they are very original and insightful, particularly in consideration of both the paucity of moderation effects in BRT works and repeated calls for detecting potential moderators in BRT literature [17]. One theoretical implication of our findings is that BRT should be updated in a more in-depth manner by theorizing boundary conditions (e.g., gender) of some BRT causal paths.

***The measurement scale of reasons for and against eWOM.*** Last but not least, the present study develops and validates the scale of reasons for and against the eWOM behavior of Millennial tourists. Cross-validation results indicate that the factor structure of the reasons scale is stable. The reasons for and against are significantly correlated with other BRT core constructs, including values, attitudes, and behaviors. This suggests that the nomological validity of our newly developed reasons scale has been successfully achieved. To our knowledge, this particular scale has not been documented elsewhere in the literature prior to this study; thus, this theoretical and instrumental contribution is very original and valuable. Future scholars should use our scale to explore more nomological networks relevant to reasons in the context of eWOM behavior of Millennial tourists.

### 5.2. Practical Implications

Our study provides marketers with many insights about Millennial tourists’ values, attitudes, reasons, and behaviors in the context of eWOM in social media. Our findings could serve as guidelines for destination managers and marketers to develop and maintain a competitive advantage. First, managers are encouraged to possess a profound understanding of tourists’ attitudes concerning eWOM behavior, as such attitudes serve as a pivotal factor in influencing the efficacy of eWOM communication. The results show that reasons for and against eWOM both have significant direct and indirect effects (via attitude) on Millennial tourists’ future eWOM behavior. Unlike prior research (e.g., [8,9]) that solely emphasizes promoting positive eWOM behavior through enhancing tourist attitudes or reasons, this study adopts a distinct perspective by focusing on tourists’ reasons and attitudes toward eWOM behavior. It advocates for the use of market research and customer feedback collection to gain insights into tourists’ attitudes concerning eWOM and the underlying reasons for their eWOM behavior. By adopting this approach, practitioners could identify and promote Millennial tourists’ positive attitudes, which in turn results in corresponding positive eWOM behavior.

Second, destination managers and marketers could use our measurement scale of reasons as a diagnostic tool that enables destinations to gain insight into tourists’ psychological perceptions and experiences of the corresponding destinations. Moreover, our findings are useful for the design of tourism products, the delivery of services, and the development of marketing strategies for Millennial tourists. For example, the results show that tourists are inclined to share primarily due to their memorable travel experiences; destinations can intensify the advancement and marketing of distinctive tourism products. In the decision-making process, unique and distinctive tourism products tailored to the preferences of specific tourists have often proven to be favorable pull motives [77]. Conversely, unsatisfactory tourism experiences, such as service failure, often lead to negative eWOM effects among tourists. This suggests that destination managers should prioritize both infrastructure construction and service quality at the destination to ensure tourists’ satisfactory experiences.

Third, it is imperative for destination stakeholders to craft gender-sensitive marketing strategies. Given the proven moderation effect of gender, integrating gender considerations into marketing strategies is crucial. This entails conducting gender-specific research to gain insights into the unique needs and preferences of both male and female Millennial tourists and devising marketing campaigns that resonate effectively with each demographic. Targeted gender-specific advertisements and offers on social media can further encourage tourists to share eWOM. In response to negative eWOM, managers must act promptly and employ gender-tailored communication strategies such as empathy for female Millennials and decisive action for their counterparts of male tourists.

## 6. Limitations and Future Research

First, the generalizability of our study findings is contingent upon specific situations. The overall measurement model of this study exhibits both configural invariance and full metric invariance across two split samples (noted earlier). This result demonstrates, as per DeVellis and Thorpe [58], the generalizability of our overall measurement model. The statistical results obtained by analyzing the overall structural model, however, are not generalizable to other samples external to Mainland China because all respondents are Chinese Millennials. Future studies should verify our study findings in other countries or regions with different national cultures. Second, the cross-sectional nature of our data suggests the nomological associations but not the causalities [78], among the BRT latent constructs included in our framework. Future studies are warranted to replicate our study by using longitudinal and/or experimental data to verify the causalities included in this study. Finally, future studies are suggested to explore more moderators besides gender within the BRT framework. This enables scholars and practitioners to understand factors affecting Millennial tourists’ behavior of eWOM in a more comprehensive and insightful manner.

## 7. Concluding Remarks

In closing, we have successfully developed and validated a measurement scale of Millennial tourists’ reasons for and against their eWOM behavior. We have taken a step further to investigate the influences of reasons as well as their interplay with other BRT theoretical antecedents, including global attitudes and self-enhancement, on existing and future eWOM behavior of Millennial tourists. Most of our research findings regarding direct, mediation, and moderation are exploratory and thus valuable and insightful, particularly considering that the roles of global attitude’s mediation and gender’s moderation in the relationships between reasons and existing/future behaviors have not been documented elsewhere in the literature prior to this study. The overall measurement model exhibits factorial invariance across our two split samples, suggesting the generalizability of the factor structure of the model according to DeVellis and Thorpe [58]. The causalities between BRT constructs remain, however, inclusive because our empirical data are cross-sectional. Future studies are warranted to replicate our study in a Western culture using longitudinal data. Generally, this study offers theoretical and practical inputs for future investigators to expand upon regarding how, why, and under what conditions and reasons affect the eWOM behavior of Millennial tourists.

## Figures and Tables

**Table 1:** Demographic profile of respondents.

Demography	Category	Number of Respondents	Percentage (%)
Age	18–23	146	25.5
	24–29	318	55.6
	30–35	108	18.9
Occupation	In full-time education	135	23.6
	In full-time employment	324	56.6
	Freelance	113	19.8
Education	High school or technical secondary school and below	68	11.9
	University or college	449	78.5
	Postgraduate and above	55	9.6
Gender	Male	367	64.2
	Female	205	35.8
Monthly income	≤USD 413.20	97	17.0
	USD 413.35–688.68	104	18.2
	USD 688.82–1377.36	289	50.5
	>USD 1377.36	82	14.3

Note: USD 1 equals CNY 7.26 approximately.

**Table 2:** Assessment results of the overall measurement model.

Constructs	Mean	SD	Loading	CR	AVE
Self-enhancement (SE)				0.830	0.550
SE1	4.25	0.783	0.749		
SE2	3.86	1.015	0.653		
SE3	4.09	0.863	0.791		
SE4	4.06	0.933	0.767		
Reasons for (RF)				0.855	0.599
RFf1	4.30	0.548	0.872		
RFf2	4.29	0.495	0.800		
RFf3	4.35	0.524	0.758		
RFf4	4.46	0.456	0.647		
Reasons against (RA)				0.812	0.694
RAf1	2.02	0.805	0.988		
RAf2	2.51	1.094	0.641		
Attitude (ATT)				0.849	0.585
ATT1	6.08	0.909	0.795		
ATT2	5.72	1.025	0.737		
ATT3	6.09	0.994	0.748		
ATT4	6.08	1.054	0.778		
Existing behavior (EB)				0.911	0.836
EB1	3.24	0.897	0.909		
EB2	3.02	0.734	0.920		
Future behavior (FB)				0.842	0.516
FB1	5.94	0.958	0.724		
FB2	5.85	1.009	0.708		
FB3	5.77	0.996	0.700		
FB4	5.89	0.992	0.742		
FB5	6.01	0.877	0.716		

Note: (1) Reasons for and Reasons against are parceling based on EFA result. (2) RFf1, 2, 3, 4 consisted of five (RF6, RF7, RF11, RF12, RF15), four (RF2, RF4, RF10, RF16), three (RF9, RF13, RF17), and two items (RF1, RF3). (3) RAf1,2 consisted of ten (RA6, RA7, RA8, RA9, RA10, RA11, RA12, RA14, RA15, RA16) and three items (RA2, RA3, RA4).

**Table 3:** Latent variable correlations.

	ATT	EB	FB	RA	RF	SE
Attitude (ATT)	0.765					
Existing behavior (EB)	0.276	0.914				
Future behavior (FB)	0.640	0.188	0.718			
Reasons against (RA)	−0.330	−0.061	−0.382	0.833		
Reasons for (RF)	0.558	0.176	0.666	−0.424	0.774	
Self-enhancement (SE)	0.522	0.279	0.481	−0.179	0.400	0.742

Note: (1) N = 572. (2) The values on the diagonal are squared AVE values. (3) Below the diagonal are correlation values, which are all statistically significant at 0.001 level. (4) Values are all obtained by using SmartPLS 3.0 software.

**Table 4:** Hypotheses testing results.

Path	Coefficients	95% Bias-Corrected Bootstrap		T-Value	*p*-Values	Decision
		Lower Bound	Upper Bound			
Direct effects						
RF→ATT (H1a)	0.370	0.260	0.477	6.596	0.000	Supported
RA→ATT (H1b)	−0.110	−0.186	−0.041	2.936	0.003	Supported
RF→EB (H2a)	−0.009	−0.124	0.108	0.158	0.874	Not Supported
RA→EB (H2b)	−0.003	−0.096	0.090	0.033	0.974	Not Supported
RF→FB (H2c)	0.360	0.248	0.473	6.350	0.000	Supported
RA→FB (H2d)	−0.085	−0.165	−0.010	2.116	0.034	Supported
ATT→EB (H3a)	0.258	0.134	0.369	4.363	0.000	Supported
ATT→FB (H3b)	0.386	0.256	0.508	5.843	0.000	Supported
SE→RF (H4a)	0.392	0.312	0.473	9.416	0.000	Supported
SE→RA (H4b)	−0.164	−0.248	−0.085	3.790	0.000	Supported
SE→ATT (H5)	0.354	0.233	0.478	5.704	0.000	Supported
Mediation effects						
RF→ATT→EB (H6a)	0.095	0.050	0.143	4.105	0.000	Supported
RA→ATT→EB (H6b)	−0.028	−0.051	−0.010	2.601	0.009	Supported
RF→ATT→FB (H6c)	0.144	0.077	0.221	3.779	0.000	Supported
RA→ATT→FB (H6d)	−0.043	−0.082	−0.013	2.337	0.019	Supported
Moderation effects						
Gender * RF→EB (H7a)	0.143	−0.058	0.357	1.336	0.182	Not Supported
Gender * RA→EB (H7b)	0.249	0.025	0.474	2.190	0.029	Supported
Gender * RF→FB (H7c)	0.173	0.001	0.338	2.040	0.041	Supported
Gender * RA→FB (H7d)	0.018	−0.163	0.183	0.214	0.831	Not Supported

Notes: (1) 572 parent samples with 5000 bootstraps enabled in SmartPLS 3.0 (2) Control variables include gender, age, income, and education. (3) * indicates moderating the relationship.

## Data Availability

The data presented in this study are available on request from the corresponding authors.

## Appendix A. Scale Items

**Self-enhancement**

(1) When I share travel experiences on social media platforms, I will feel a sense of accomplishment.
(2) When I share travel experiences on social media platforms, I will have a chance to get the reward.
(3) When I share travel experiences on social media platforms, I can increase the recognition of others.
(4) When I share travel experiences on social media platforms, other people will perceive me as knowledgeable.

**Attitude**

(1) I think sharing travel experiences on social media platforms is a good idea.
(2) I think sharing travel experiences on social media platforms is a wise move.
(3) I think sharing travel experiences on social media platforms is valuable to me.
(4) I like sharing travel experiences on social media platforms.

**Existing eWOM behavior**

(1) After this trip, the number of times you have already shared this travel experience on social media platforms is: 0 1 2–3 4–5 more than 5 times
(2) Please specify the social platforms on which you share content. Wechat Weibo Tiktok Little red book Others

**Future eWOM behavior**

(1) I will post or share images of the destination on social media platforms.
(2) I will talk about the destination with others on social media platforms.
(3) I will reply to the comments about the destinations.
(4) I will say things about the destination on social media platforms.
(5) I will provide recommendation to others to visit the destination on social media platforms.

## Appendix B. Items from Qualitative Interviews and Literature Review (N = 25)

**Table:**

Constructs and items	Mean Value	Source	
		References	Interview
**Reasons for**			
RF1. The destination has beautiful natural attractions.	4.55	Tasci et al., 2021 [79]	√
RF2. The destination has a comfortable climate.	4.45	Abubakar & Mavondo, 2014 [80]	√
RF3. The destination has unique cultural characters.	4.30		√
RF4. The destination has good transport infrastructure.	3.80		√
RF5. The destination has a dynamic tourism atmosphere.	4.05		√
RF6. The destination’s employees offer good service.	4.00	Abubakar & Mavondo, 2014 [80]	√
RF7. The destination has hospitable locals.	4.15	Tasci et al., 2021 [79]	√
RF8. The destination is socially safe and made me feel secure.	3.95		√
RF9. The destination fulfills my expectations.	4.25	Kalinić et al., 2019 [81]	√
RF10. The destination offers a wide variety of cuisines.	4.20		√
RF11. My sharing can provide reference information for other tourists.	4.05	Alexandrov et al., 2013 [82]	√
RF12. My sharing can record my traveling experience.	3.90		√
RF13. The destination made me feel at ease.	4.35	Abubakar & Mavondo, 2014 [80]	√
RF14. I came across new things at the destination.	4.30	Chen et al., 2020 [83]	√
RF15. I enjoyed a unique experience at the destination.	4.45	Chen et al., 2020 [83]	√
RF16. I highly recognized the destination.	4.35		√
RF17. I liked the local customs and culture.	4.35		√
**Reasons against**			
RA1. The destination has too many visitors.	3.60	Abubakar & Mavondo, 2014 [80]	√
RA2. There was some pollution at the destination (e.g., beaches, streets, etc.).	3.95	Kim, 2022 [84]	√
RA3. The general local infrastructure of the destination was not adequate (e.g., public toilets, car parks, etc.).	3.90	Kim, 2022 [84]	√
RA4. The overall quality of services of the destination was poor.	3.85		√
RA5. The destination was not prompt in responding to my complaint.	3.85	Yadav et al., 2023 [85]	√
RA6. *The destinations faired to offer adequate nightly entertainment.	2.85		√
RA7. Tourism destinations were falsely advertised (e.g., landscapes, services, etc.).	3.80		√
RA8. Little few choices of hotels and poor accommodations at the destination.	3.45		√
RA9. The service providers at the destination failed to protect my personal information.	3.55		√
RA10. I was cheated and overcharged by businesses at the destination.	3.90	Kim, 2022 [84]	√
RA11. The destination existed forced consumption.	3.95	Kim, 2022 [84]	√
RA12. The destination is not able to provide relevant information (e.g., accommodation, transport, etc.).	3.45	Yadav et al., 2023 [85]	√
RA13. Don’t want more tourists to fall for scams.	3.90		√
RA14. I was generally dissatisfied with the trip.	3.85	Yang et al., 2024 [86]	√
RA15. *I don’t like to share my life on social media platforms.	3.15		√
RA16. *I’m not willing to advertise a destination for free	2.70		√
RA17. *There has been a lot of similar sharing on social media platforms	3.05		√

Note: * indicates deleted items in Experts’ review.

## Appendix C. Items from Experts’ Review. (N = 20)

**Table:**

Constructs and items	Source	
	References	Interview
Reasons for		
RF1.The destination has beautiful natural attractions.	Tasci et al., 2021 [79]	√
RF2.The destination has a comfortable climate	Abubakar & Mavondo, 2014 [80]	√
RF3.The destination has unique cultural characters.		√
RF4.The destination has good transport infrastructure.		√
RF5.*The destination has a dynamic tourism atmosphere.		√
RF6.The destination’s employees offer good service.	Abubakar & Mavondo, 2014 [80]	√
RF7.The destination has hospitable locals	Tasci et al., 2021 [79]	√
RF8.*The destination is socially safe and made me feel secure.		√
RF9.The destination fulfills my expectations	Kalinić et al., 2019 [81]	√
RF10.The destination offers a wide variety of cuisines.		√
RF11.My sharing can provide reference information for other tourist	Alexandrov et al., 2013 [82]	√
RF12.My sharing can record my traveling experience.		√
RF13.The destination made me feel at ease.	Abubakar & Mavondo, 2014 [80]	√
RF14.*I came across new things at the destination.	Chen et al., 2020 [83]	√
RF15.I enjoyed a unique experience at the destination.	Chen et al., 2020 [83]	√
RF16.I highly recognized the destination.		√
RF17.I liked the local customs and culture.		√
Reasons against		
RA1.*Sharing would take me lots of time and energy	Lee, 2009 [87]	√
RA2.I worry that my sharing will be ignored	Liu et al., 2020 [88]	√
RA3.I worry that my sharing will be used in a way I did not foresee.	Liu et al., 2020 [88]	√
RA4.I worry that my sharing will be controversial.	Liu et al., 2020 [88]	√
RA5.*The destination has too many visitors.	Abubakar & Mavondo, 2014 [80]	√
RA6.There was some pollution at the destination (e.g., beaches, streets, etc.).	Kim, 2022 [84]	√
RA7.The general local infrastructure of the destination was not adequate (e.g., public toilets, car parks, etc.).	Kim, 2022 [84]	√
RA8.The overall quality of services of the destination was poor.		√
RA9.The destination was not prompt in responding to my complaint.	Yadav et al., 2023 [85]	√
RA10.Tourism destinations were falsely advertised (e.g., landscapes, services, etc.).		√
RA11.Little few choices of hotels and poor accommodations at the destination.		√
RA12.The service providers at the destination failed to protect my personal information.		√
RA13.*I was cheated and overcharged by businesses at the destination.	Kim, 2022 [84]	√
RA14.The destination existed forced consumption.	Kim, 2022 [84]	√
RA15.The destination is not able to provide relevant information (e.g., accommodation, transport, etc.).	Yadav et al., 2023 [85]	√
RA16.Don’t want more tourists to fall for scams.		√
RA17.*I was generally dissatisfied with the trip.	Yang et al., 2024 [86]	√

Note: * indicates deleted items in EFA.

## Appendix D. EFA Result. (N1 = 287)

**Table:**

Factor	Factor Loading	Rotation Sum of Squared Loading		
		Total	% Variance	% Cumulative
Factor 1: Service failures (α = 0.918)		5.875	21.761	21.761
RA12	0.773			
RA9	0.764			
RA14	0.758			
RA15	0.753			
RA10	0.749			
RA6	0.737			
RA8	0.725			
RA7	0.700			
RA11	0.688			
RA16	0.647			
Factor 2: Memorable travel experiences (α = 0.661)		2.516	9.318	31.079
RF15	0.715			
RF11	0.668			
RF6	0.553			
RF7	0.442			
RF12	0.429			
Factor 3: Accessibility and cuisines (α = 0.667)		2.321	8.598	39.677
RF16	0.730			
RF10	0.702			
RF4	0.542			
RF2	0.526			
Factor 4: Side effects of sharing (α = 0.653)		2.260	8.372	48.049
RA3	0.823			
RA2	0.809			
RA4	0.800			
Factor 5: Happy feelings (α = 0.653)		1.624	6.017	54.065
RF13	0.688			
RF9	0.573			
RF17	0.566			
Factor 6: Natural and cultural attractions (α = 0.572)		1.621	6.004	60.069
RF1	0.739			
RF3	0.638			
